# Unifying the gestural and the vocal in the evolution of culture, the arts, and the brain

**DOI:** 10.3389/fpsyg.2026.1706986

**Published:** 2026-03-03

**Authors:** Steven Brown

**Affiliations:** Department of Psychology, Neuroscience & Behaviour, McMaster University, Hamilton, ON, Canada

**Keywords:** acting, cultural evolution, motor learning, social learning, the arts, vocal learning

## Abstract

Cultural evolution in humans is based on the transmission of knowledge and know-how through the process of social learning. Humans have evolved two distinct mechanisms of social learning, although they tend to be discussed in completely separate literatures. They are gestural (or motor) learning and vocal learning. Within the arts, gestural learning is important for the evolution of dance and mime, while vocal learning is important for the evolution of oral literature and vocal music. These two learning systems get jointly recruited to mediate the process of impersonation during theatrical role playing; an actor has to depict both the gestural and vocal features of a portrayed character. An evolutionary synthesis of gestural and vocal learning undergirds the human capacity for culture, including the arts. I discuss potential brain mechanisms for this synthesis in which the neural pathways for the gestural and the vocal may converge.

## 1 Two forms of cultural learning: the gestural and the vocal

Humans have achieved a highly sophisticated form of culture (Boyd and Richerson, 2005; Mesoudi, 2011; Richerson et al., 2016). This is characterized not only by a complexification and diversification of material culture (e.g., tools, infrastructure), but by a comparable complexification of social organization, leading to the large-scale societies of modern times (Turchin, 2013). The human capacity for culture involves not only the ability to maintain traditions across generations (Jagiello et al., 2022), but also the talent for generating novelty although acts of creativity (Csikszentmihalyi, 1988; Fogarty et al., 2015; Carr et al., 2016), as seen in the striking acceleration in product innovation across all domains of technology in modern times (Wilf, 2015).

Cultural evolutionists tell us that the most important mechanism that enables culture is *social learning*, which is the ability to faithfully transmit information and/or objects from person to person both across and within generations via processes such as imitation, emulation, and teaching (Boyd and Richerson, 1985, 2005). Social learning from others is contrasted with individual-level (or asocial) learning, such as trial-and-error learning, which is thought to be far less efficient at maintaining and transmitting information across individuals (Boyd and Richerson, 1985; Mesoudi, 2011). Culture depends on transmission processes that transcend what any one individual could either learn on their own or pass on to individuals in a single generation. This is aided in humans by the explicit practice of teaching, in which experts pass on information and know-how to novices (Csibra and Gergely, 2011; Gärdenfors, 2017), although teaching practices are widespread in the animal world (Hoppitt et al., 2008). Because of social learning, individuals do not have to “reinvent the wheel” each generation, but can instead inherit technical knowledge from their predecessors. This allows this knowledge to be transmitted faithfully across generations. This process can result in progressive changes to technologies over time, a phenomenon referred to as “cumulative culture” (Tomasello et al., 1993; Tennie et al., 2009; Dean et al., 2014) (See Whiten, 2019 for a discussion of cumulative culture in non-human animals).

A key point that is not discussed in virtually all presentations about the evolution of culture is that humans have evolved not one but *two* distinct imitative mechanisms for social learning: gestural (or motor) learning and vocal learning. Gestural/motor learning (hereafter gestural learning) provides an important basis for praxis and the transmission of cultural knowledge and skills. Imitative learning of this type has been implicated in both the production of tools and the use of tools during human evolution (Csibra and Gergely, 2011; Wynn and Coolidge, 2014; Gärdenfors, 2017; Stout and Hecht, 2023). Cultural evolutionists emphasize the fact that when children are asked to imitate the actions of an adult, they imitate the *trajectory* of the modeled action – even task-irrelevant features of the action – and not merely the endpoint of the action, where the latter is referred to as emulation (Tennie et al., 2009). Such high-fidelity imitation is considered to be an important cognitive and behavioral substrate for both the vertical and horizontal transmission of information about how to work with tools and with the functional objects that they act on. Gestural imitation, aside from its role in skill learning, underlies the process of social conformity that is viewed by cultural evolutionists as being a strong driving force for the evolution of cooperation in humans (Henrich and Boyd, 1998; Mesoudi and Lycett, 2009).

The other major route for social learning is the learning of communication sounds through vocal imitation, permitting the *oral transmission* of cultural information that is acquired through speech and music (or their combination), including social norms, stories, proverbs, prayers, and songs. Vocal imitation is important not just for the learning of speech and music during childhood development (Kuijpers, 1987), but also for the ability of adults to produce imitations of people’s voices, the sounds of nature, and inanimate sounds in order to convey information about these things to other people (Aboitiz, 2012; Brown, 2017). The signals that are acquired through vocal learning in both humans and non-human vocal learners communicate information about the external world, individual and group identity, social relationships, and the contexts of social interactions (Brenowitz and Beecher, 2023). Importantly, vocal learning is a more complex skill than auditory learning alone – such as when a dog learns to sit in response to the human imperative statement “sit” – since it requires the vocal capacity to replicate what is heard.

The imitative learning of communication sounds is thought to allow communication systems to develop a greater level of both complexity (e.g., larger sound repertoires) and context flexibility (e.g., voluntary control) compared to the non-learned vocal-communication systems of most animals (Mercado et al., 2014; Carouso-Peck et al., 2021; Janik and Knörnschild, 2021; Allen et al., 2022; Brenowitz and Beecher, 2023; Arnon et al., 2025). It may also allow for a vocal exaggeration of body size (Ravignani and Garcia, 2022). Vocal learning is rare in animals, being found in less than a dozen unrelated clades, among them songbirds, humpback whales, and some bat species (Petkov and Jarvis, 2012; Vernes et al., 2021). Some vocal learners acquire a single song early in development, whereas others, such as humans, are lifelong vocal learners capable of engaging in vocal imitation throughout the lifespan (Petkov and Jarvis, 2012). This includes the ability to mimic the sounds of other species, such as when parrots mimic human speech or when humans mimic parrots mimicking human speech.

**Figure 1:**
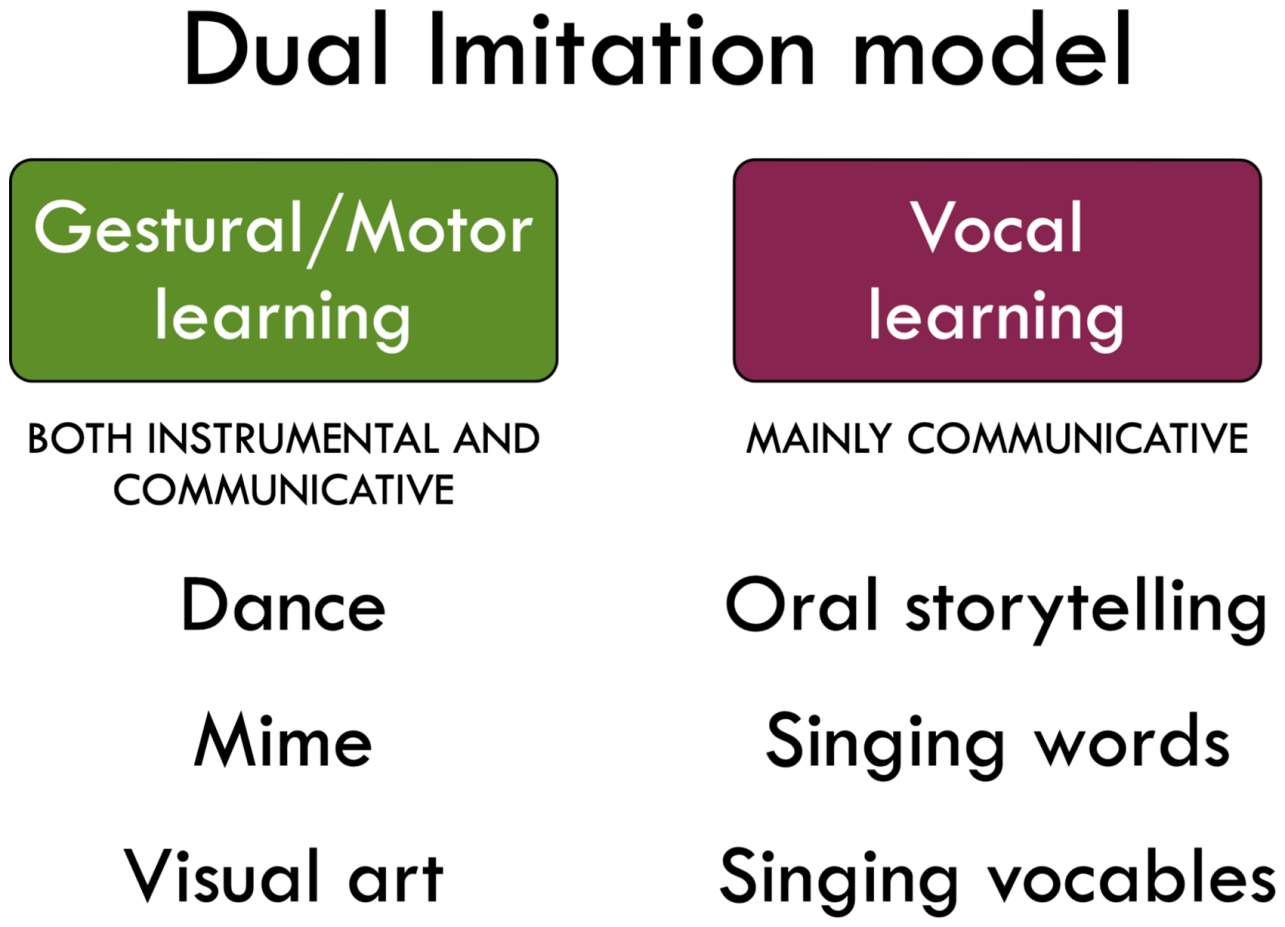
A dual imitation model. Humans have evolved two forms of social learning that underlie the capacity for culture. The lower part of the figure lists artforms that are acquired through either gestural learning or vocal learning on their own.

One of the key points of this article is that gestural learning and vocal learning have generally been discussed independently of one another in completely separate literatures, one focused on the evolution of praxis and tool use (gestural learning) and the other on the evolution of vocal communication and language (vocal learning). In order to rectify this, I present a Dual Imitation perspective for the origin of human culture that seeks to unify these two domain-specific routes to social learning (Figure 1). An important point of distinction between the two is that vocal imitation serves a primarily communicative function; it is employed for the learning of acoustic communicative systems, namely speech and music in humans. However, gestural imitation has a dual functionality that is both instrumental and communicative. The standard literature on social learning in humans focuses overwhelmingly on the instrumental manifestation of gestural learning, most especially for transmitting information about the manufacture and use of tools (Tennie et al., 2009; Mesoudi, 2011; Tehrani, 2011; Stout and Chaminade, 2012; Stout and Hecht, 2023). However, gestural learning can be the basis for communicative functions as well. For example, the emergence of gesturing during childhood is seen by developmental psychologists as the first form of linguistic communication (Bates and Dick, 2002), although some evidence suggests that the earliest forms of gesturing may more deictic than symbolic (Burkhardt-Reed et al., 2025), In addition, gestural models of the origin of language posit that a pantomimic precursor served as the earliest form of linguistic communication and that this was later replaced by vocal communication (Hewes, 1973; Armstrong and Wilcox, 2007; Tomasello, 2008; Aboitiz, 2012; Arbib, 2012). Modern-day derivatives of this system include sign languages in deaf communities (Taub, 2004), writing systems (Rogers, 2005; Powell, 2012), and the gesticulations and pointing gestures that accompany speech among hearing people (McNeill, 2005). The gestural route is thus more multifaceted than the vocal route. Vocal learning is far less associated with the types of instrumental actions – such as technology development and cumulative improvement – that are common in the gestural realm, although, as mentioned below, the spoken command “Alexa, turn on the lights” represents a situation where a learned vocalization can serve as a replacement for an instrumental hand action. Imperatives might in fact be the most instrumental of speech acts.

Imitation is the sensorimotor process of motorically recreating what one perceives (Heyes, 2021). Therefore, gestural and vocal learning require domain-specific sensorimotor systems to accomplish this perception/action matching. More specifically, gestural imitation depends on the visual observation of motor actions, followed by their reproduction by the viewer using the same effectors that the model used in producing the observed action. By contrast, vocal imitation depends on the auditory perception of invisible vocalizations and their reproduction by the vocal-motor system of the listener, including both phonation (pitch) and articulation (phoneme quality). The exception to this invisibility may be times when we are able to observe someone’s mouth movements as they are vocalizing and thus use this motion as a visual cue to aid in imitation, most especially when it comes to articulation.

The key point here is that there is every reason to believe that the neural systems underlying gestural and vocal imitation are distinct and domain-specific such that they should be independently impacted by disease states or lesions (Dresang et al., 2023). At the same time, there is evidence that the capacities for gestural and vocal imitation might develop in tandem in infants (Masur and Ritz, 1984) and that they may be impacted jointly by disease states, such as in the case of autism spectrum disorder (Espanola Aguirre and Gutierrez, 2019). In fact, phylogenetic evidence suggests that the neural pathways for gestural and vocal communication may have co-evolved during human evolution (Hecht et al., 2025). This is supported by developmental evidence in humans that speech and symbolic gesturing emerge in tandem. As Charman (2006) noted, “nonverbal gestures develop hand-in-hand with verbal communication skills” (p. 98), and that “vocal and gestural imitation are both longitudinally associated with language development” (p. 111). Finally, at a theoretical level, Donald (1991, 2013) proposed that there was a stage of hominin evolution that he called Mimetic Culture that was characterized by a complex suite of imitative behaviors that integrated gestural and vocal (though non-linguistic) actions, including collective dancing and singing.

## 2 The arts: the gestural and the vocal

Can we apply the insights of the Dual Imitation perspective to the arts? Can we talk about the gestural and the vocal in the arts? In the next section, I will argue that theatrical acting is a unification of the gestural and the vocal in the arts. However, I first want to focus on artforms that engage *either* the gestural *or* the vocal on their own (see Figure 1). To a first approximation, we can think about this topic in terms of artistic-production skills that require either gestural or vocal learning in order to be acquired by practitioners. Learning to perform the choreography of a dance requires gestural learning, whereas learning to sing a song or recite an epic requires vocal learning. Let us thus consider examples such as these that emphasize one route or the other to social learning, but not their combination.

### 2.1 Gestural learning

The previous section talked about the fact that, while vocal learning serves a primarily communicative function for humans, gestural learning can be done for either instrumental or communicative purposes. In talking about the gestural arts in this section, we come face to face with the communicative and expressive aspect of gesture, although instrumentality is present in the visual arts and instrumental forms of music production. Dance is perhaps the canonical example of an arts domain that is acquired through gestural learning. Dances vary extensively with regard to the joints that are most active in producing their movement patterns (Lomax, 1968; Brown, 2022). Some dances recruit unusual joints in their movements. In Flamenco dancing, for example, the wrist and fingers are active joints, while in Indian Kathak dancing, the eyes and neck are active effectors of movement. Therefore, each genre of dance provides unique challenges to dancers in learning how to achieve fluid movement at its active joints, including producing these movements in a rhythmic manner where timing is specified.

People learn to produce these movement and rhythmic patterns by imitating role models. In Western culture, there are dance instructors who create regimens for teaching dance movements to novices through demonstration and the presentation of corrective feedback in a personalized manner. In traditional cultures, this appears to be much less common. Dances are far more likely to be learned implicitly by observing and imitating role models in the absence of personalized instruction or error correction by these experts. This is because dances in traditional cultures are typically components of religious rituals in which engaging in the dance movement is often more important than performing the “correct” choreography. As a result, there are most likely no “dance classes.” A person seems to learn the movement patterns of a dance by simply engaging in the dance repeatedly over time and attempting to correct errors on the fly through imitative observation of expert role models, although this process is insufficiently studied in the ethnographic literature on dance.

Another facet of imitation in dance, aside from the learning process itself, relates to group dances that are done in unison where everyone performs the same movements at the same time, as in many ring dances and line dances. I have referred to such processes of matching other people’s movements as “acting like” other people (Brown, 2025). Each dancer imitatively matches the movement patterns of the other dancers in the group, although the movements themselves generally have to be pre-learned in order for such imitation to occur seamlessly. Donald (1991) refers to coordinated rituals of this kind in which people match their actions to one another as being forms of “group mimesis,” highlighting the inherently imitative nature of these behaviors.

A common convention of dance in Western culture is that dancers do not vocalize. While there are exceptions to this convention, such as in musical theatre, dance is typically considered to be a *mute* artform that prioritizes the gestural over the vocal. Another such artform is mime theatre. Whereas dance-forms can be either narrative or abstract with regard to their content, mime theatre is a narrative form of body movement in which the mime conveys a story and often depicts characters, but does so in a voiceless manner. The mime conveys narrative meaning through pantomimic gestures, rather than speech. This non-vocality is shared with narrative forms of dance, such as ballet. However, the narrative dancer, in contrast to the mime actor, takes advantage of a musical score that serves as a richly expressive acoustic cue that can counterbalance the non-vocal nature of the artform and convey emotional meanings acoustically through the use of scales and musical-prosodic cues. In addition, in traditional forms of dance in indigenous cultures, dancers often use body percussion as a means of making their dance movements audible, for example through the use of leggings or rattles. Occasionally, such body percussion is vocal in nature, such as the grunting sounds produced by Maori warriors in their dances, although their vocalizations include chanted words as well (Youngerman, 1974). Mime theatre, by contrast, is not only mute but is generally silent as well. It should be noted that, at the origins of mime theatre in ancient Rome, the pantomime was a dancer who was accompanied by instrumentalists and a singer (Hall, 2008, 2013), a practice that has changed dramatically in contemporary times. Outside of the arts, other silent forms of communication that depend on gestural/motor learning include sign language and writing.

Visual art is a third type of artform that is purely gestural. Visual art, unlike dance and mime theatre, makes extensive use of tools and media for the creation of art objects (Boas, 1927). The learning process for a visual artist is not about movement patterns per se, but about acquiring the *instrumental skills* needed to work with tools and to apply them to media, such as using a brush to apply paint to a canvas. In addition, while drawing can be achieved through memory representations of the depicted object, scene or person, it often occurs in the presence of the depicted model, such as during the painting of a portrait. This is another level at which imitation is operative in visual art beyond skill learning per se, namely in creating a visual *copy* of a model (Brown, 2022). Finally, while dance and mime are done in the context of a performance, the act of generating products in visual art is typically done outside of a public performance, with the exception of modern practices of “performance art” (Goldberg, 2011). Generally speaking, visual art is disseminated to audiences via exhibition, rather than performance, although the two might function comparably when it comes to processes of public display of the artworks (Brown, 2022).

### 2.2 Vocal learning

The second grouping of artforms shown in Figure 1 is comprised of vocal arts, including oral storytelling and vocal music. In traditional cultures, people learn stories, poems, epics, aphorisms, and songs by imitating people who know how to perform these works through a process of vocal learning. For example, individuals learn stories by hearing people recite them, such as during evening storytelling sessions in indigenous cultures (Wiessner, 2014). In Western culture, parents tell bedtime stories to their children, and likewise teach them nursery rhymes and children’s songs (e.g., *Twinkle Twinkle*). In the best of cases, a good storyteller is also a good actor and will embody the voice and gestures of the characters during the sections of dialogue in the story (Matharu et al., 2021). However, storytelling can also be accomplished quite readily without this, as in a poetry recitation. To the extent that storytelling does indeed include character portrayal and acting, then it is discussed in the following section about role playing.

The other purely vocal artform shown in Figure 1 is singing, which can occur either with or without words (Sachs, 1943; Lawson, 2023). When singers create melodies using nonsense syllables alone, it is referred to as vocable singing (Boas, 1927), and this includes humming. As with oral storytelling, singing can also occur in a theatrical manner in opera and musical theatre, where the performers are not only singers but actors as well. We will focus here on singing outside of the context of theatre, hence emphasizing the vocal in the absence of the gestural. The learning of acoustic patterns in music can also be achieved through the use of musical instruments that function as surrogates for the voice and that often mimic the timbre and prosodic features of the voice, such as stringed and aerophone instruments. In many cases, this vocal surrogacy is carried out using manual gestures in the body, such as when a person plays the piano, where the instrument serves as a tool. The same is true of instrumental surrogates for speech, such as the “talking drums” of drummed languages (Stern, 1957; Arhine, 2009). In such situations, *gestural learning replaces vocal learning* in the production and replication of communication sounds.

It is difficult to conceive of the reverse situation where vocal learning replaces gestural learning in the acquisition of motoric skills, although imperative speech-acts such as “Alexa, turn on the lights” might be a modern-day manifestation of the voice replacing the hands during an instrumental task. Likewise, in rally car driving, the co-driver navigates the vehicle through verbal communication with the driver, another example of the use of imperative speech-acts. An unusual example of inter-species communication is the use of a specialized vocal sound by human honey hunters in Africa directed at honeyguide birds, which signals to the birds that the humans want to be led to bees’ nests (Spottiswoode et al., 2016), another possible example of an imperative sound.

Finally, another facet of imitation in the vocal arts, aside from the learning process itself, relates to group choruses that are done in unison such that everyone performs the same melodic line at the same time. In such contexts, each singer imitatively matches the melody (and key) of the other singers in the group, although the parts themselves generally have to be pre-learned in order for such imitation to occur seamlessly.

## 3 Gestural + vocal → role play

Having described the gestural and the vocal in distinct branches of the arts, let us now examine artforms that combine them. While there are multiple interactions between the gestural and the vocal in the arts – for example, singers like Elvis Presley who accompany themselves on instruments, or singers like Madonna who dance while singing – the focus here will be placed on theatre as the dominant format. The theatrical arts are characterized by the fact that performers engage in role playing to impersonate people whom they themselves are not. Acting requires that performers think about *both* the gestural and the vocal side of their portrayal. As a result, it is a unification of the two. When an actor impersonates a character in a theatrical work, they have to depict not only that person’s voice, but their style of movement, their body expression, and their facial expression. In certain artforms, the vocal part can be sung, rather than spoken. This can occur either throughout the work (as in opera) or in particular sections of it (as in musical theatre). Likewise, in certain artforms, the actors are not humans, but are instead visual representations of them, as in puppet theatre, animation, and role-playing video games. Elsewhere (Brown, 2024, 2025), I have described how the narrative arts in general, including the theatrical arts, were derived from the evolution of the human capacity for pantomime as a gestural means of communicating information about people, perhaps incorporating vocalization as well (Żywiczyński et al., 2018; Zlatev et al., 2020).

Role playing should not be thought of as a third-person means of *describing* people – as in visual art –but instead as a first-person means of *embodying* them through acts of impersonation and pretense. Actors present themselves in social settings as people whom they themselves are not. Acting theorists tell us that there are two principal means by which an actor is able to enter into a character: a gestural route and a psychological route (Kemp, 2012). In gestural acting, the actor aims to create the surface impression of being a character without directly experiencing the psychological states of the character. For example, a gestural actor would depict an anguished character by conveying the external features of an anguished person’s expressions, but without feeling anguish him/herself. This process connects theatre technique with dance traditions cross-culturally (Barba, 1995). By contrast, in psychological acting, the actor attempts to “become” the character by developing the psychological dispositions – beliefs, motivations, and emotions – and thus the felt experience of the character in the moment. Much of this is oriented toward developing an understanding of why the character is motivated to do what they are doing at any given moment in the narrative. Psychological acting is associated with the writings of the Russian acting theorist Stanislavski during the first half of the 20th century, although the debate about whether an actor should create a surface illusion of a character or instead transform themselves into the character was widely discussed in the 18th century.

The French philosopher Diderot encapsulated this debate in his book *The Paradox of the Actor*, written in 1773 but published in 1830. Diderot discussed the relative costs and benefits of the gestural vs. psychological approaches to character portrayal in actors. He himself took the perspective that not only does an actor not feel a character’s emotions in performance but that s/he most definitely *should not* feel these emotions. He believed that feeling the emotions of a character would have a negative impact on the portrayal, and that it would make performances idiosyncratic and uneven across repeated presentations of the work. For him, the talent of the actor “depends not, as you think, upon feeling, but upon rendering so exactly *the outward signs of feeling* that you fall into the trap” (Diderot, 1830/2019:16, emphasis added). Stanislavki (1936/1989) took the opposite perspective, arguing that an actor needs to directly experience the emotions of the character during a performance, and not simply mimic them: “The great actor should be full of feeling, and especially *he should feel the thing he is portraying*. He must feel an emotion not only once or twice while he is studying his part, but to a greater or lesser degree every time he plays it” (p. 14, emphasis added). In other words, an actor should live the part. He “must fit his own human qualities to the life of this person, and pour into it all of his own soul” (p. 15).

Given that acting is a union of the gestural and the vocal, what is the relationship between the two during performance? Do they function independently or do they work synergistically in a mutually reinforcing manner? Berry et al. (2022) carried out an experimental study in which trained actors were tasked with reciting a fixed text while portraying nine different stock characters in different trials. The characters varied along the two orthogonal personality dimensions of assertiveness and cooperativeness (Berry and Brown, 2017), and the data analysis sought to identify the main effect of each dimension. Parallel behavioral analyses were carried out for body expression, facial expression, and vocal prosody, where the first two were measured using 3D motion capture. Berry et al.’s main observation was that there was *correlated expression* across the body, face, and voice during character portrayal, resulting in a trimodal synthesis. More specifically, they found that raising the pitch of the voice or increasing vocal loudness was correlated with vertical raising of the head relative to the chest and a greater degree of jaw lowering in the face. Head raising and jaw lowering are, in fact, physiological requirements for placing the vocal tract in the appropriate configuration for generating high-pitched and loud vocalizations, and so these results might be expected. However, the results with the body were more surprising. In particular, raising the pitch of the voice or increasing vocal loudness was correlated with both vertical raising and horizontal widening of the arms, much like a person assuming the posture of a victory pose. In accordance with these results, it was shown that expansion of the arms was correlated with raising of the head and lowering of the jaw, creating an expansive pose across the body.

Overall, the study of Berry et al. (2022) provided evidence of correlations between gestural expression and vocal expression – including an interesting arm/voice connection – during character portrayal in trained actors, where both reflect the personality traits of the portrayed characters. Role playing is thus an important unification of the gestural and the vocal in the arts. The gestural and the vocal reinforce one another during character portrayal in the theatrical arts, just as they do during everyday emotional expression. The work of Berry et al. also supports previous studies that have shown correlated patterns between vocal acoustics and patterns of body and/or facial movement. For example, Scherer and Ellgring (2007), in a study of multimodal expression, observed correlated changes between the voice and face for several basic emotions, including correlations of both high pitch and loudness with activity in the brow, cheek, and jaw. Some multimodal studies of facial expression in the context of vocal production have focused on singing (Thompson and Russo, 2007; Livingstone et al., 2015), where correlations have been observed between vocal pitch and both raising of the brow and lowering of the jaw, and some have focused on speech (Scherer and Ellgring, 2007; Livingstone et al., 2015).

## 4 The gestural and the vocal in the brain

I conclude this article with a discussion about whether there might be shared neural resources for gestural and vocal imitation in the brain. To think about this, we have to consider the concept of “somatotopy” in neuroscience or the notion of a “homunculus” in the motor cortex (Penfield and Boldrey, 1937). While many parts of the human nervous system are thought to have a somatotopic organization – whereby different effectors of the body are organized in an orderly manner across the region – there is also evidence that such maps might be less discrete than was originally conceived, instead containing regions of overlap that blur the distinction between various effectors (Graziano, 2016; Gordon et al., 2023). Evidence for somatotopy has been found in motor-related regions like the primary motor cortex, supplementary motor area, cingulate motor area, cerebellum, and basal ganglia, among others. In such regions, there is reasonable evidence for spatial maps that distinguish among the three principal body regions of the orofacial effectors, the upper limbs, and the lower limbs.

While it is conceivable that the hand and voice might overlap in any or all of these regions, I would like to place my focus on the precentral gyrus (PCG) of the posterior frontal lobe. While the dorsal part of the PCG consists mainly of primary motor cortex – corresponding with area 4 in the cytoarchitectonic scheme of Brodmann areas (BA) – the ventral part of the PCG is a hybrid. The posterior part is primary motor cortex (BA 4), but the anterior part is premotor cortex (BA 6). Beyond this cytoarchitectonic difference alone, there is an interesting *somatotopic* difference between these two regions. The primary-motor part of the ventral PCG is orofacial. It contains representations for the larynx, lips, jaw, tongue, and pharyngeal muscles (Loucks et al., 2007; Brown et al., 2008; Simonyan et al., 2009; Bouchard et al., 2013; Conant et al., 2014; Dichter et al., 2018; Eichert et al., 2020; Liang et al., 2023). However, the premotor part that is directly anterior to it is completely different. It is a hand-movement area that is responsive to the visual observation of hand actions (Bremmer et al., 2001; Caspers et al., 2010; Papitto et al., 2020). This area, called PMv (for ventral premotor cortex), is one of the core regions of the mirror system of the human brain (Hamilton, 2015). It shows joint responsiveness to motor activity and action observation. This visuo-manual area sits directly anterior to the orofacial motor cortex in the ventral PCG, creating a curious juxtaposition between the gestural and the vocal in the brain.

A neural underpinning of the Dual Imitation model of human cultural evolution needs to account for how visual information reaches hand-motor areas (for gestural imitation), as well as how auditory information reaches vocal-motor areas (for vocal imitation). As mentioned earlier, imitation is nothing if not a motoric recreation of what is perceived. Let us consider some of the neural pathways for imitation. For gestural imitation, PMv is thought to receive visual information from the posterior parietal cortex. One of the key inputs comes the anterior part of the intraparietal sulcus, called area AIP (Rizzolatti and Luppino, 2001; Bufacchi et al., 2023). This is considered to be another key node of the mirror system in both monkeys and humans (Hamilton, 2015). The projection from AIP to PMv in humans most likely occurs via the third branch of the superior longitudinal fasciculus (SLF), so-called SLF III. An important caveat about PMv’s role in imitation comes from the observation that studies that have compared imitation tasks against a matched non-imitative movement condition – rather than a low-level baseline condition – do not show PMv activation, but instead activity in the supramarginal gyrus (SMG) and inferior frontal gyrus (IFG) (Chaminade et al., 2002; Decety et al., 2002; Koski et al., 2003). Hence, the SMG and IFG may show more specificity for imitation than does PMv. However, the pathway from the SMG to the inferior frontal gyrus is most likely via SLF III as well.

Looking now to vocal imitation, the major pathway of interest is the arcuate fasciculus (AF), which sends projections from the posterior part of the temporal lobe, including auditory areas, to premotor areas in the inferior frontal region (Catani et al., 2007; Dick et al., 2014; Fernández-Miranda et al., 2015). The AF constitutes part of the “dorsal stream” of the speech network that is important for “translating acoustic speech signals into articulatory representations in the frontal lobe” (Hickok and Poeppel, 2007), hence audio-vocal integration. The terminations of the AF are highly disputed in humans. While classic models place the terminations exclusively in the IFG (BA 44 and 45), others also include more-posterior terminations in the premotor part of the PCG in BA 6 (Bernal and Altman, 2010). According to the latter model, PMv would be a termination of the AF, in addition to more-anterior terminations like Broca’s area (or its homologue in the right hemisphere).

**Figure 2:**
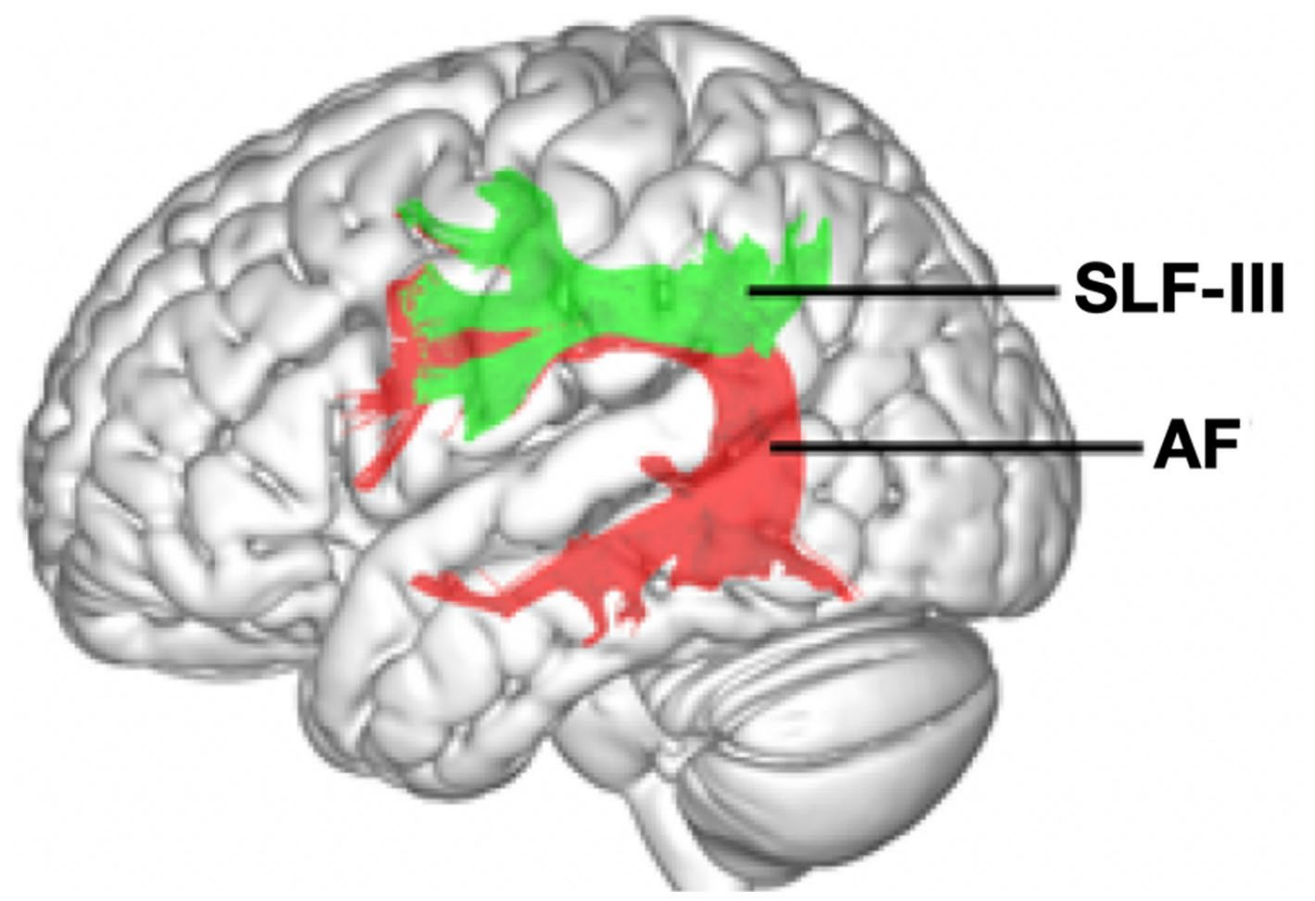
Tractography data showing potential overlap between the arcuate fasciculus (AF, red) and the third branch of the superior longitudinal fasciculus (SFL III, green) in the region of the precentral gyrus and extending into the inferior frontal gyrus. Reproduced with permission from Zhao et al. (2023).

Another topic of dispute about the AF, aside from its frontal terminations, is *whether it is a component of SLF III*. This article is not the appropriate place to discuss the evidence for or against this, but it is important to consider the implications of the contention that the AF is a component of SLF III. A clinical tractography study by Zhao et al. (2023) demonstrated the general overlap between SLF III and AF in the region of the anterior PCG (see Figure 2). If this is indeed the case, then it might suggest that the PCG is a potential *convergence point* for the pathways that mediate gestural imitation (SLF III) and vocal imitation (AF). I will refer to this speculative hypothesis as the Shared Arcuate model. While Figure 2 depicts this with regard to terminations in the PCG, the model also applies comparably to terminations that extend into the that extend into the IFG, which is the standard conception of the termination region of the AF and SLF III. The main point here is that arcuate projections, whether to the PCG or IFG, potentially constitute a nexus point for the two human-specific and domain-specific imitation systems.

An fMRI study that contrasted vocal pitch imitation with non-imitative pitch production revealed activation in the larynx motor cortex and basal ganglia (Belyk et al., 2016). I am not aware of any studies that have compared speech imitation with a matched non-imitative speech condition, although Sörös et al. (2006) compared vowel imitation against non-vocal mouth movements and obtained activation in frontopolar regions. So, at this point, we can only speculate that the PCG (including PMv) and IFG are shared between gestural imitation and vocal imitation, and that the Shared Arcuate model might account for this. Much work will be needed to test this hypothesis. Such a model would jibe with the observation of “inter-effector” areas in mid-region of the PCG (Gordon et al., 2023). It also supports the contention that the ventral part of the SMG, also referred to as Spt by some researchers (Hickok and Poeppel, 2007; Buchsbaum and Esposito, 2008; Hickok, 2009), is jointly implicated in manual and phonological processing. Finally, this view is consistent with Aboitiz’s (2012) claim that the manual and vocal systems of the brain “make use of overlapping circuits” (p. 8), thereby contributing to multimodal communication.

A Shared Arcuate model has important evolutionary implications for the human capacity for culture. The AF has shown a progressive expansion in both size and complexity in moving from monkeys to chimpanzees to humans (Rilling et al., 2008). While the AF is almost always discussed in terms of vocal imitation alone, the results of Zhao et al. (2023), as well as other findings that suggest that the AF is a component of SLF III, indicate that the expansion of the AF in humans may have impacted not only vocal imitation but gestural imitation as well via the SLF III projection from AIP to PMv, or comparably from SMG to IFG more ventrally. So, the Shared Arcuate model is not only a means of uniting gestural and vocal imitation neuroanatomically, but a means of uniting them *evolutionarily* as well.

The evolutionary expansion of the AF might have enhanced domain-specific sensorimotor connections required for both gestural imitation and vocal imitation. A tractographic study in captive chimpanzees provides phylogenetic support for this contention. Hecht et al. (2025) identified microstructural properties of the AF that correlated jointly with gestural and vocal communication in their cohort of animals. These properties were shown to be associated with individual differences in *both* gestural requests for unreachable food items *and* vocal attention-getting sounds that are used in either grooming contexts or to get the attention of humans. While the Shared Arcuate model is highly speculative, it has the advantage of being parsimonious in that expansion of a single white-matter pathway during human evolution may have had a joint impact on the two domain-specific social-learning systems that have undergirded the evolution of culture in humans (see Aboitiz, 2012 for a related argument about the evolution of communication).

It should be pointed out that there is a separate literature that attempts to relate vocal learning not to gestural learning but instead to the capacity to rhythmically synchronize body movements to auditory beats, such as during dancing. This speech/dance model invokes connections between the AF and SLF in its argumentation, as in the present article. However, the hand projection in this model is not to PMv, but instead to the primary hand area in the dorsal part of the primary motor cortex (Patel, 2021). In addition, one critique that I would raise is that a model relating vocal imitation to dance should more likely involve the legs, rather than the hands.

## 5 Conclusion

The human capacity for culture is predicted on two distinct forms of social learning: gestural and vocal. This idea forms the basis of my Dual Imitation perspective, since gestural learning and vocal learning are typically discussed in completely separate literatures, one focused on the evolution of praxis and tool use (gestural learning) and the other on the evolution of vocal communication (vocal learning). With regard to the arts, there are artforms that emphasize the gestural (e.g., dance), others that emphasize the vocal (e.g., song), and some that unite the two (e.g., theatre). In particular, the impersonation of characters during theatrical role playing is a ubiquitous unification of the gestural and the vocal across human cultures. An experimental study (Berry et al., 2022) demonstrated that gestural expression and vocal expression tend to be mutually reinforcing during acting, and that they jointly reflect the personality traits of the portrayed characters.

I discussed a speculative Shared Arcuate model in which the domain-specific neural pathways for gestural and vocal imitation come together in the ventral premotor cortex via a potential convergence between SLF III and AF in the PCG and/or IFG. The AF has shown extensive expansion in humans compared to non-human primates, and so this expansion may have given rise to both gestural learning and vocal learning as newly-evolved human traits, despite the very different sensorimotor mechanisms that underlie these social-learning systems. Tractographic work in chimpanzees reveals that the microstructural properties of the AF are related to individual differences in both gestural and vocal communication (Hecht et al., 2025). In sum, the Dual Imitation perspective discussed here argues that models of the evolution of the human capacity for culture need to give joint consideration to both of the domain-specific systems of social learning that humans have evolved, rather than either one on its own. I propose that role playing is an evolved communicative behavior in humans that reflects the joint contribution of these two social-learning mechanisms. It is a marriage of the gestural and the vocal.

## Data Availability

The original contributions presented in the study are included in the article/supplementary material, further inquiries can be directed to the corresponding author/s.
